# Function of metabolic and organelle networks in crowded and organized media

**DOI:** 10.3389/fphys.2014.00523

**Published:** 2015-01-21

**Authors:** Miguel A. Aon, Sonia Cortassa

**Affiliations:** Department of Medicine, School of Medicine, Johns Hopkins UniversityBaltimore, MD, USA

**Keywords:** enzyme kinetics, metabolism, quinary structures, cytoskeleton, molecular crowding, fractal, Sierpinsky sponge, percolation

## Abstract

(Macro)molecular crowding and the ability of the ubiquitous cytoskeleton to dynamically polymerize–depolymerize are prevalent cytoplasmic conditions in prokaryotic and eukaryotic cells. Protein interactions, enzymatic or signaling reactions - single, sequential or in complexes - whole metabolic pathways and organelles can be affected by crowding, the type and polymeric status of cytoskeletal proteins (e.g., tubulin, actin), and their imparted organization. The self-organizing capability of the cytoskeleton can orchestrate metabolic fluxes through entire pathways while its fractal organization can frame the scaling of activities in several levels of organization. The intracellular environment dynamics (e.g., biochemical reactions) is dominated by the orderly cytoskeleton and the intrinsic randomness of molecular crowding. Existing evidence underscores the inherent capacity of intracellular organization to generate emergent global behavior. Yet unknown is the relative impact on cell function provided by organelle or functional compartmentation based on transient proteins association driven by weak interactions (quinary structures) under specific environmental challenges or functional conditions (e.g., hypoxia, division, differentiation). We propose a qualitative, integrated structural–functional model of cytoplasmic organization based on a modified version of the *Sierspinsky–Menger–Mandelbrot sponge*, a 3D representation of a percolation cluster, and examine its capacity to accommodate established experimental facts.

It seems to me that cells leave very little to random processes and that they have evolved the capacity to escape much of the chaos of solutions, (…). It appears unlikely that a messy alphabet soup would be used to spell out the elaborate prose of intermediary metabolism.

*James S. Clegg, 1984*

## Introduction

Cells are very far from random mixtures of molecules. The classical experiments of Kempner and Miller (1968) showed that cells are not bags of freely floating enzymes. Using cells from the unicellular eukaryote Euglena as “centrifuge tubes” (Clegg, 1984a), these authors (Kempner and Miller, 1968) could distinguish several layers within cells (that remained viable) after centrifugation. No macromolecules could be detected in the “soluble phase” and many of the main enzymes considered to exist free in solution were instead associated with layers containing organelles (mitochondria, lysosomes, nucleus) and subcellular structures (ribosomes).

Ideas about cytoplasmic organization have a long history that can be traced back to the notion of protoplasm as the substratum of cellular activity (Welch and Clegg, 2010). Early microscopic techniques (reviewed in Aon and Cortassa, 1997, Chapter 6) and the more recent cryoelectron tomography (Medalia et al., 2002) unveiled the overall crowded nature of the cellular cytoplasm populated by complex macromolecular assemblies besides subcellular organelles (Luby-Phelps, 2000; Minton, 2001; Grunewald et al., 2003; Ovádi and Norris, 2013) (see **Figure 2A**). Studies starting in the twentieth and well into the twenty-first centuries used advanced molecular–cellular biology methods including fluorescence recovery after photobleaching, in conjunction with confocal microscopy and time-resolved anisotropy methods, to establish a decrease in the diffusion coefficients of proteins in the cytoplasm and in the endoplasmic reticulum lumen compared with water (Luby-Phelps, 2000; Verkman, 2002; Rivas et al., 2004). The consequences of these results are very significant for cell function because diffusion of solutes and macromolecules in cellular compartments mediates many physiological processes, including metabolism and signaling events. On the other hand, active transport via motor proteins leads to a significantly higher mobility compared to diffusion processes (Fakhri et al., 2014).

Obtaining information about the molecular properties of proteins in the living cell is becoming an active field of research (Wirth and Gruebele, 2013). Existent and new molecular techniques and methodologies enable monitoring of native protein activity and folding in cells, offering information on concentration, dynamics, location, interactions and protein proximity (Diekmann and Hoischen, 2014; Fakhri et al., 2014).

## Enzymatic reactions in organized crowded media

Supramolecular organization and crowding are two main traits of the intracellular milieu (Aon and Cortassa, 1997; Aon et al., 2001). The cellular cytoplasm contains (macro)molecules: a mixture of molecules of low (e.g., ATP, glutathione, NADH) and higher molecular weight (e.g., proteins, lipids, polysaccharides) and macromolecular arrays (e.g., tubulin and F-actin polymers, glycogen granules), at concentrations such that they occupy a large fraction of its total volume. Such media are “crowded” but no individual (macro)molecular species is present at a high concentration *per se* (Minton, 1997).

“Background” species concern (macro)molecules that do not interact specifically with either the reactants or products of a particular reaction. Proteins in the crowded cellular environment can stick to each other through non-specific interactions (e.g., electrostatic, hydrophobic). “Background” species can also contribute large steric repulsive forces in crowded environments (Minton, 2000) that may not be observed directly because they do not lead to the formation of complexes (Zhou et al., 2008). The excluded volume effect depends on the size and concentration of molecular crowders and refers to the volume between, e.g., a pair of interacting proteins, that cannot be occupied by a third protein (Wirth and Gruebele, 2013). If the size of a (macro)molecule is comparable to the size of background species, the available volume is considerably smaller (i.e., excluded volume higher) than in the case that the (macro)molecule is relatively tinier. Lower available volume increases the contribution of steric repulsion to reduce entropy and increase free energy (Rivas et al., 2004). If we consider that to maximize the available volume is a way to reduce free energy then (macro)molecular crowding facilitates a decrease in excluded (occupied) volume via, e.g., molecular compaction and association (Minton, 2000, 2001; Ellis, 2001). Another consequence of crowding is that, via decrease of excluded volume, the folded over the unfolded state of a protein or protein complex is favored (Wirth and Gruebele, 2013).

The reaction rate of an enzymatic reaction may be controlled at diffusional (substrate(s), *S*, access to the enzyme's, *E*, active site) and/or kinetic (an intrinsic step in the reaction scheme limits the rate) levels. Broadly speaking, diffusional and kinetic control may be assessed through diffusion, percolation or transport of the species *S* and *E* involved, and *k*_*2*_, the rate constant of the enzyme–substrate, *ES*, complex transformation into product according to the Henri–Michaelis–Menten (HMM) formalism (Segel, 1975).

For conceptual purpose, let us consider a simple reaction converting *S* into *P*, that involves one ligand, *S*, one catalytic site, *E*, and one enzyme–substrate complex, *ES* (Aon et al., 2004b):

*Diffusion, percolation, transport of S and/or E*

(1) $$\to \;\to \;\to S\;+\;E\overset{k_{1}}{\underset{k_{-1}}{\leftarrow \;\to }}\;ES\overset{k_{2}}{\to }E\;+\;P$$

*k*_*2*_, also known as the catalytic rate constant, *k_cat_*, and *k*_−*1*_ are monomolecular rate constants whereas *k*_*1*_ is a bimolecular rate constant. In the derivation of the HMM equation from a quasi–steady-state assumption, the dynamics of the *ES* complex association–dissociation is considered to be so fast that its concentration can be treated as if it were in steady state. Accordingly, the *k*_*1*_ step is considered not to be limiting the *S* → *P* conversion. Importantly, from experiments performed over the past two decades, single-molecule enzymology has provided insights into how specific enzymes—particularly molecular motors and nucleic acid enzymes—work at the molecular level. These studies confirmed that the HMM mechanism expressed in Equation (1) holds at the single molecule level (Xie, 2013).

In heterogeneous, organized, media the rate of encounter between *E* and *S* may be subjected to transport restrictions generated by anomalous diffusion. Protein stickiness will increase the apparent viscosity of the cytoplasm thus decreasing the diffusion coefficient, D, since both (viscosity *vs*. D) are inversely related (Dix and Verkman, 2008). Anomalous diffusion introduces a time dependence in D, essentially due to the medium heterogeneity (Wirth and Gruebele, 2013) (see below Section Fractal Kinetics in Organized Crowded Media). Both translational and rotational diffusion can be influenced by the excluded volume and the shape of the crowding protein more than other factors such as hydrodynamic or direct interactions (Balbo et al., 2013). Thus, molecular crowding can affect *k*_*1*_ decreasing enzymatic rates, essentially because as higher the excluded volume by (macro)molecules the higher the rate limitation of the *E*–*S* encounter (Homchaudhuri et al., 2006; Pastor et al., 2014). In support of this interpretation, enzymatic reactions occurring in the presence of increasing dextran concentrations exhibited lower V_max_ and higher K_M_, the Michaelis–Menten constant (Pastor et al., 2014). The volume occupied by dextran, independent of its size, had an important role on the initial velocity of the hydrolysis of N-succinyl-L-phenyl-Ala-p-nitroanilide catalyzed by alpha-chymotrypsin (Pastor et al., 2011). The K_M_ increase could be attributed to a slower diffusion of the protein due to the presence of crowding, whereas the decrease in V_max_ could be explained by the effect of mixed inhibition by product, which is enhanced in crowded media (Pastor et al., 2014). These results also underscore the relevant role of enzyme size in the initial velocity of reactions occurring in dextran crowded media. When enzymes are small the reaction's initial velocity mainly depends on the excluded volume. However, for large enzymes, the initial velocity of the reaction is also affected by the size of obstacles present in the environment.

Modeling and experimental work (Kim and Yethiraj, 2009; Pastor et al., 2014) also supports the idea that macromolecular crowding can contribute significantly to changes in enzymatic reactions (Vasilescu et al., 2013). To simulate rapid metabolite transfer between the enzymatic components of the phosphotransferase system (PTS), macromolecular crowding had to be assumed both to increase the association rate constants and to decrease the dissociation rate constants of the PTS complexes (Rohwer et al., 1998). However, crowding was not necessary to simulate yeast glycolysis suggesting that in this eukaryote it does not affect the glycolytic pathway (referred in Rivas et al., 2004). Cortassa and colleagues have also shown that, at least for two glycolytic enzyme couples, the effects of tubulin cytoskeleton proteins was specific and independent from crowding (Cortassa et al., 1994).

Very recent studies using carbon nanotubes for intracellular tracking of kinesin-1 motility highlight stirring dynamics as another important mode of active intracellular transport. Recorded kinesin-1 motility in COS-7 cells over five orders of magnitude in time (Fakhri et al., 2014) enabled the detection of different dynamic regimes ranging between the extremes of random thermal diffusion and kinesin-driven directed transport propelled by stirring dynamics as a non-equilibrium regime between those extremes.

Weak interactions can mediate transient protein–protein interactions collectively known as “quinary structures,” a term introduced by McConkey (1982) to define a fifth level (beyond the quaternary) of inherently transient protein structural organization. Thermodynamically, the transience of a quinary structure is based on the low stability of the molecular interaction as well as the low energetic barrier between molecular states (Wirth and Gruebele, 2013). Quinary structure has been implicated in a number of cellular processes from metabolism [e.g., the metabolon (Srere, 1987), the protein synthesis pathway (Dang et al., 1985)] to cell signaling (Li et al., 2012). Its inherent transience facilitates dynamic spatial organization of macromolecules in the cytoplasm via loose groupings of, e.g., proteins, when they are working together, but not otherwise (Wirth and Gruebele, 2013).

Overall, (macro)molecular crowding can drive molecular associations in the cytoplasm, and via modulation of the available volume or transient quinary structures influence the kinetics of biochemical reactions.

## Cytoskeleton organization and metabolic fluxes

The intracellular environment is not only highly crowded but exhibits a high degree of dynamic organization governed by the principles of self-organization, as they apply to thermodynamically open non-equilibrium systems such as cells (Nicolis and Prigogine, 1977; Aon and Cortassa, 1997; de la Fuente, 2013). By exchanging energy, matter or information with their environment cells or tissues can exhibit emergence that is they self-organize their internal structure and dynamics with novel and sometimes surprising macroscopic properties. For example, the ubiquitous cytoskeletal protein network (actin or tubulin) behaves as a non-linear dissipative system, i.e., it consumes adenine nucleotides to polymerize, with the ability to self-organize, e.g., oscillate (Mandelkow et al., 1989; Mandelkow and Mandelkow, 1992; Tabony and Job, 1992) or alternate in a bistable manner (Aon et al., 1996b; Aon and Cortassa, 1997) between polymerized and depolymerized states. Structurally, the cytoskeletal network exhibits fractal properties (Mandelbrot, 1982; Feder, 1988), i.e., spatially organized in a self-similar manner thus exhibiting an alike form when observed at different degrees of magnification (Rabouille et al., 1992; Aon and Cortassa, 1994; Losa and Nonnenmacher, 1996).

The highly dynamic polymer composite of the cellular cytoplasm is dominated by protein polymers, e.g., microtubules, F-actin and intermediate filaments. In axons in the spinal cord the interaction between neurofilaments and F-actin results in a gel with particular viscoelastic properties (Leterrier et al., 1996). The nucleotide triphosphate hydrolysis-driven polymerization–depolymerization dynamics of cytoskeletal proteins is reciprocally influencing and being influenced by the myriad of reactions involved in intracellular transport (e.g., motors), metabolism, cell locomotion, and muscle contraction among other functions. For example, microtubule tracks are embedded in the viscoelastic actin cytoskeleton, which in turn fluctuates as a result of stresses generated by cytoplasmic myosins; myosin locally contracts the actin network with an attachment time of several seconds, followed by sudden release (Fakhri et al., 2014).

Cytoskeleton organization is also influenced by cytoplasmic molecular crowding because it favors protein interactions that may form modular complexes (Spirin and Mirny, 2003); some of these molecular complexes constitute stable or transient multienzyme associations (metabolon) (Srere, 1987) capable of metabolic channeling (Welch, 1977; Ovádi and Srere, 2000). In metabolic channels, reactions are facilitated by product–substrate transfer between closely associated enzymes (Ovádi and Srere, 2000).

**Figure 1:**
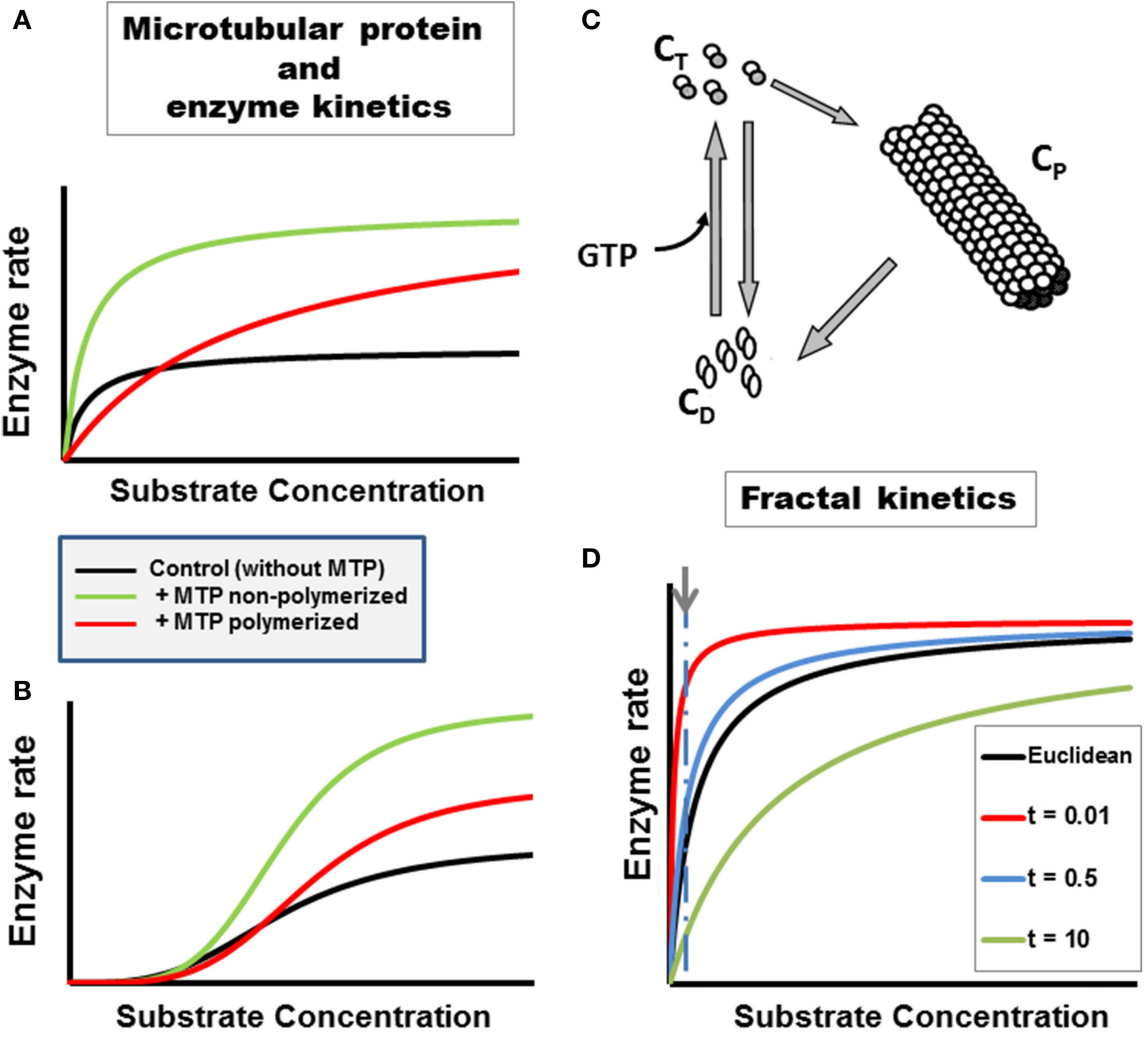
**Enzyme kinetics in heterogeneous, organized fractal medium**. The concentration and polymeric status of the cytoskeleton components tubulin and microtubule associated proteins, i.e., microtubular protein (MTP), can modulate metabolic fluxes and the kinetics of enzymatic reactions through various mechanisms (Aon and Cortassa, 1997). Examples with two enzymatic couples, glucose 6 phosphate dehydrogenase coupled to hexokinase (G6PDH/HK, **A**):
$$\begin{array}{l} \text{ Glucose }+\text{ ATP}\overset{\text{HK}}{\to }\text{Glucose }6\text{ P }+\text{ ADP}\\ \text{Glucose }6\text{ P }+\text{ NADP}\overset{\text{G6PDH}}{\to }6\text{ phospho-gluconate }+\text{ NADPH} \end{array}$$ or pyruvate kinase coupled to lactate dehydrogenase (PK/LDH, **B**):
$$\begin{array}{l} \text{Phosphoenolpyruvate }+\text{ ADP}\overset{\text{PK}}{\to }\text{Pyruvate }+\text{ ATP}\\ \text{ Pyruvate }+\text{ NADH}\overset{\text{LDH}}{\to }\text{Lactate }+\;{\text{NAD}}^{+} \end{array}$$ Microtubular protein (MTP) in the flux-stimulatory range (1 mg/ml) (Cortassa et al., 1994) was added to the assay medium containing either G6PDH/HK **(A)** or PK/LDH **(B)**. The substrate concentration of the limiting enzyme, NADP or phosphoenolpyruvate (PEP) for HK/GGPDH or PK/LDH couples, respectively, was varied. The presence of polymerized or non-polymerized brain MTP elicited changes in kinetic parameters: **(A)** In the presence of polymerized MTP, G6PDH exhibited an eight-fold increase in the K_M_ for NADP and a two-fold increase in V_max_ with respect to the control without MTP; non-polymerized MTP only induced a two-fold increase in V_max_ without changing the K_M_. **(B)** In the presence of polymerized brain MTP, PK exhibited an increase in cooperativity and in V_max_ with respect to controls as a function of PEP whereas non-polymerized MTP induced an even higher increase in cooperativity and V_max_. **(C)** The cycle of assembly–disassembly of MTP. In this cartoon, the cycle of polymerization–depolymerization of MTP assumes that tubulin may exist in one of three forms: polymerized (C_P_), non-polymerized bound to GTP (C_T_) or bound to GDP (C_D_). A model of the MTP cycle and its effects on PK kinetics is presented in Aon and Cortassa (1997). **(D)** Michaelis–Menten kinetics in fractal medium. The dependence of the initial rate of a canonical enzymatically-catalyzed reaction as a function of its substrate is displayed. The *h* parameter, reflecting the characteristics of the medium, e.g., obstacle density, and the time-dependence of kinetic constants was calculated as described in Aon et al. (2004b) (see also Section Fractal Kinetics in Organized Crowded Media). The time points, *t*, at which the simulations were performed are those indicated in the symbol legend (in min). For simulating reactions occurring in Euclidean space, the parameter values were identical to those of fractal medium except that the rate constants were time-independent (see Aon et al., 2004b for calculation details). At short times the reaction rate becomes much larger, at low *S* levels, in fractal than in Euclidean medium (indicated by arrow and dashed line). As time passes the reaction rate becomes, transiently, slower in fractal than in Euclidean space; the maximal rate being identical in both cases though achieved at larger *S* in fractal medium.

Microtubules, actin microfilaments and intermediate filaments represent an enormous protein surface in the cell with an estimated area of 3000 μm^2^ for a typical mammalian cell in culture (Luby-Phelps, 2000). Thus, the cytoskeleton provides an interface for binding a variety of proteins and enzymes. The binding affinity depends on several factors, including phosphorylation status (Luther and Lee, 1986; Roberts and Somero, 1987; Pedrotti et al., 1996), the presence of other proteins, e.g., Microtubule Associated Proteins (MAPs) (Cortassa et al., 1994; Aon and Cortassa, 1997), or enzymatic activity (Cortassa and Aon, 1994; Vertessy et al., 1997; Cassimeris et al., 2012). Protein binding also affects microtubule and enzyme dynamics (Aon et al., 2001; Ovádi and Norris, 2013; Olah et al., 2015). The enhancement of metabolic flux depends upon several factors: (i) the presence for some enzymatic reactions of MAPs apart from tubulin; (ii) the concentration of microtubular protein (MTP); and (iii) the polymeric status (Cortassa et al., 1994). For example, an increase in flux through pyruvate kinase coupled to lactate dehydrogenase was elicited by MTP in a particular concentration range (Cortassa et al., 1994; Aon et al., 1996a) (Figures 1A–C; see figure legend for details). Paclitaxel and nocodazole, two drugs affecting microtubule organization and dynamics in opposite ways, were able to alter the secretion of proteolytic enzymes associated with invasion and metastasis of tumor cells (Alonso et al., 1999). While paclitaxel promotes microtubules polymerization, nocodazole elicits de-polymerization and as such they enhanced or reduced, respectively, the secretion of the urokinase-type plasminogen activator and the matrix metalloproteinase 9 to the culture medium in F3II mammary-carcinoma cells (Alonso et al., 1999).

Ultrasensitivity is a more sensitive response than the one expected from the classical hyperbola of Michaelis–Menten kinetics (Goldbeter and Koshland, 1982; Koshland et al., 1982). The “normal” hyperbolic response requires an 81-fold change in ligand (e.g., substrate, effector) to increase the reaction rate from 10 to 90% of the maximal velocity. Thus, ultrasensitive systems are those that need less than 81-fold change whereas sub-sensitive ones demand more. Depending on their polymeric status, cytoskeletal protein dynamics (actin, MTP) can modulate the ultrasensitive response of enzymatic systems (Aon et al., 2001). This modulation can be mediated by the cytoskeleton in the cell stress response involving changes in volume due to osmotic regulation (Busch et al., 1994; Haussinger et al., 1994a,b; Aon et al., 2000b).

More recent studies show that the heterodimer tubulin composed of α and β subunits can selectively modulate the mitochondrial outer membrane (MOM) permeability via the voltage-dependent anion channel (VDAC) (Rohwer et al., 1998; Rostovtseva and Bezrukov, 2012) thus having an impact on cellular and mitochondrial energetics (Guzun et al., 2011; Gonzalez-Granillo et al., 2012; Rostovtseva and Bezrukov, 2012). Tubulin in the nM range can influence the voltage sensitivity of VDAC reconstituted into planar phospholipid membranes, and ADP availability to the adenine nucleotide translocator in isolated mitochondria (Rostovtseva et al., 2008). Indeed, VDAC (or porin) is the most abundant protein in the MOM and is primarily involved in the ATP/ADP exchange between the cytoplasm and mitochondria (Rostovtseva and Colombini, 1997; Colombini, 2004).

## Fractal kinetics in organized crowded media

The impact of self-organized cytoskeletal proteins in fractal forms upon the dynamics of cellular biochemistry started to be explored more than 20 years ago. Studying chemical reactions in heterogeneous media, Kopelman (1988) made two crucial observations: (i) reactions proceed faster in disconnected (shredded) topologies than in connected ones, and (ii) the rate constants become time-dependent, scaling with time as *t*^−*h*^; the *h* parameter reflects the characteristics of the medium, e.g., obstacle density, through the fractal dimension, and provides a link with the kinetic rate constant (Kang and Redner, 1984; Dewey, 1995; Aon et al., 2004b). In a medium like the cellular cytoplasm, molecular diffusion becomes anomalous and diffusion coefficients time-dependent due to heterogeneity given not only by (macro)molecular crowding but also by rheological changes impinging on viscosity (Forgacs and Newman, 1994; Aon and Cortassa, 1997).

Aon and Cortassa (1994) and Forgacs (1995) proposed that the cellular cytoplasm is organized as a percolation cluster. It was further conjectured that as a highly shredded object, a percolation cluster may, in principle, bestow more catalytic power to cytoplasmic enzymes. Indeed, medium organization in percolation clusters may enhance reaction rates at short times (Aon et al., 2004b; Hiroi et al., 2011) (Figure 1D). The kinetics of biochemical reactions in the cytoplasm depend on (macro)molecular crowding given by the cytoskeleton organization. Hiroi et al. (2011) showed that changing the reaction rate may be possible when the degree of intracellular macromolecular crowding is modified by experimentally manipulating the structure of the cytoskeleton. Cytoskeleton disruption with cytochalasin B and colchicine changed the anomalous diffusion parameter exhibited by enzymes and substrates/products in the cellular cytoplasm. Since the total protein concentration was maintained this suggested that not merely the concentration of intracellular proteins, but also their physiological organization profoundly affects diffusion of free molecules in a cell (Hiroi et al., 2011).

In cellular biochemistry, the fractal approach has been directed to understanding the organization and behavior of (macro)molecules in cells (Rabouille et al., 1992; Savageau, 1995; Liebovitch and Todorov, 1996; Aon et al., 2004b; Schnell and Turner, 2004; Aon and Cortassa, 2009). The approach to fractal kinetics in cells differs between authors. The dependence of the rate constant upon *h* has been modeled according to a fractal (Zipf–Mandelbrot) distribution (Schnell and Turner, 2004); assuming HMM kinetics in 2D lattices using Monte Carlo simulations with time-dependent rate constant (Berry, 2002), or in terms of the dependence of the parameter *h* on the spectral dimension, *D*s, for HMM or sigmoidal kinetics (Aon et al., 2004b; Hiroi et al., 2011). Main findings show that: (i) spatial segregation of substrate and products increase with the degree of obstruction in the medium making stronger the deviation of the rate constants at longer times and, consequently, the fractal kinetic description as compared with the classical approach (Berry, 2002; Schnell and Turner, 2004); (ii) at short times the reaction rate becomes much larger, at low substrate levels, in fractal than in Euclidean space (Figure 1D); this behavior depends on the time-dependence of the K_M_, or an increase in cooperativity and reaction amplification in allosteric kinetics (Aon et al., 2004b). The quickly relaxing molecular mechanisms, when cells are challenged by sudden changes in environmental conditions, would provide fast and precise adaptation. Indeed, fast responses can lead to slow exhaustion processes preventing lack of substrates, effectors for reactions locally, as found in fractal media organized like percolation clusters (Hiroi et al., 2011).

## Computational modeling of cytoplasmic structure–function

Interactivity in complex spatiotemporally organized systems like the cellular cytoplasm is fundamental to their counterintuitive behavior and one of the main reasons justifying the need of mathematical modeling for their study. What we seek to understand is how function is coordinated in a cell that exhibits spatially distributed heterogeneous and compartmentalized subsystems with simultaneously unfolding dynamics (Aon, 2013). Factually, (macro)molecular crowding and cytoskeleton organization (i.e., structural) are able to influence the dynamics of biochemical reactions (i.e., functional), yet how does the structural–functional coupling unfolds in physiologically meaningful spatiotemporal patterns is far from clear. One reason is that computational modeling has been mainly concerned with structural or biochemical networks but not with their integrated function. However, forced by computational burden, modeling of cytoplasmic structure–function faces some daunting challenges, beyond the complexity of the task, that demand choices between atomistic molecular-dynamic simulations (McGuffee and Elcock, 2010; Mereghetti and Wade, 2012) and lower resolution coarse-grained models (Moore et al., 2014). Mesoscale models represent a reasonable trade-off between higher simplicity (e.g., treating macromolecules as single interacting centers) while amenable to include finer biopolymer representations to address mustiscale problems of diffusion and interaction, as recently reported for the *Escherichia coli* cytoplasm (Trovato and Tozzini, 2014). Modeling can help decide quantitative issues such as whether moderate attraction between proteins and crowding molecules, on the order of 1 kJ/mol, can counteract the excluded volume effect (Rosen et al., 2011) or which macromolecule sizes will experience the strongest attraction and anomalous diffusion (Trovato and Tozzini, 2014).

Some insight into how structural dynamics can affect biochemical function comes from computational modeling of MTP dynamics coupled to the glycolytic pathway and its branches to the Krebs cycle, ethanolic fermentation, and the pentose phosphate (PP) pathways. This study showed that MTP dynamics can coordinately increase or decrease the flux through glycolysis, and that depending on the degree of MTP polymerization a negative control may be exerted by the PP pathway on glycolysis (Aon and Cortassa, 2002). These results may be relevant for cancer therapy because the PP pathway is critical for tumor cells to generate intermediates for nucleic acid synthesis and provide NADPH required both for the synthesis of fatty acids and cell survival under oxidative stress (Patra and Hay, 2014).

A key for progress in this complicated research field will be to adopt an experimental–modeling synergy involving iteration of the loop: simulation–validation and prediction–experimentation (Cortassa and Aon, 2013).

## Emergence in subcellular organelle networks

Cytoplasmic organization comprises not only biochemical reactions but also organelles representing membrane-bound subcellular compartments. Depending on their specific function and situation, subcellular compartments such as mitochondria can play substantial roles in physiology as well as pathophysiology. In heart muscle, for example, mitochondria appear as a network in the form of a regular lattice, spanning the whole myocardial tissue like a power grid (Slodzinski et al., 2008; Aon et al., 2009). Certainly, other cytoplasmic organelles can be subject to the principles of self-organization such as the nucleus and the Golgi complex (Misteli, 2001) as well as the genome itself (Misteli, 2009), but this possibility needs experimental support.

Self-organized collective dynamics in cardiac myocytes arise from synergistically coupled subcellular networks of, e.g., mitochondria (Aon et al., 2006b) or Ca^2+^ release units, the latter constituted by four compartments (sarcoplasmic and junctional reticulum, myoplasmic and dyadic space) (Nivala et al., 2012). Emergence in these networks occurs through signaling via second messengers such as reactive oxygen species (ROS) or Ca^2+^. In addition to the normal excitation–contraction–metabolism coupling, a rich dynamic spectrum results, including oscillations, electrical or chemical waves, action potential duration alternans, early or delayed after depolarizations among others (Aon et al., 2004a; Zhou et al., 2010; Nivala et al., 2012; Qu, 2013; Zorov et al., 2014).

In heart mitochondria it was found that the transition from physiological to pathophysiological behavior happens as an emergent phenomenon of the cardiac mitochondrial network, with all the characteristics of systems at critical state (Aon et al., 2006b). This transition occurs at the percolation threshold as determined by applying percolation theory (Aon et al., 2004a). Percolation describes how local neighbor–neighbor interactions among elements in a lattice can scale to produce a macroscopic response spanning from one to the other end of a mitochondrial array (Stauffer and Aharony, 1994). Such a “spanning cluster” forms when there is a critical density of elements close to the threshold for a transition (the percolation threshold). A mitochondrial percolation cluster attains criticality at a certain threshold level of ROS (Aon et al., 2004a). The transition is self-organized, occurs with all the traits of universality—that is, with similar critical exponents as predicted by percolation theory (Schroeder, 1991; Stauffer and Aharony, 1994; Sornette, 2000), and the mitochondrial cluster exhibits fractal organization (Aon et al., 2004b). The ensuing collective oscillations—which involve at least 60% of the mitochondrial network—are synchronized by ROS via ROS-induced ROS release (Zorov et al., 2000, 2014; Aon et al., 2003; Brady et al., 2006) through a diffusion-based mechanism (Zhou et al., 2010).

In yeast, spontaneous oscillations of *Saccharomyces cerevisiae* mitochondrial redox states and membrane potential occur within individual yeasts (Aon et al., 2007b), and synchrony of yeast population indicates the operation of an efficient system of cell–cell interaction to produce concerted metabolic multicellular behavior (Murray et al., 2003, 2013; Lloyd and Murray, 2006, 2007; Roussel and Lloyd, 2007).

## Toward an integrated structural–functional model of cytoplasmic organization

**Figure 2:**
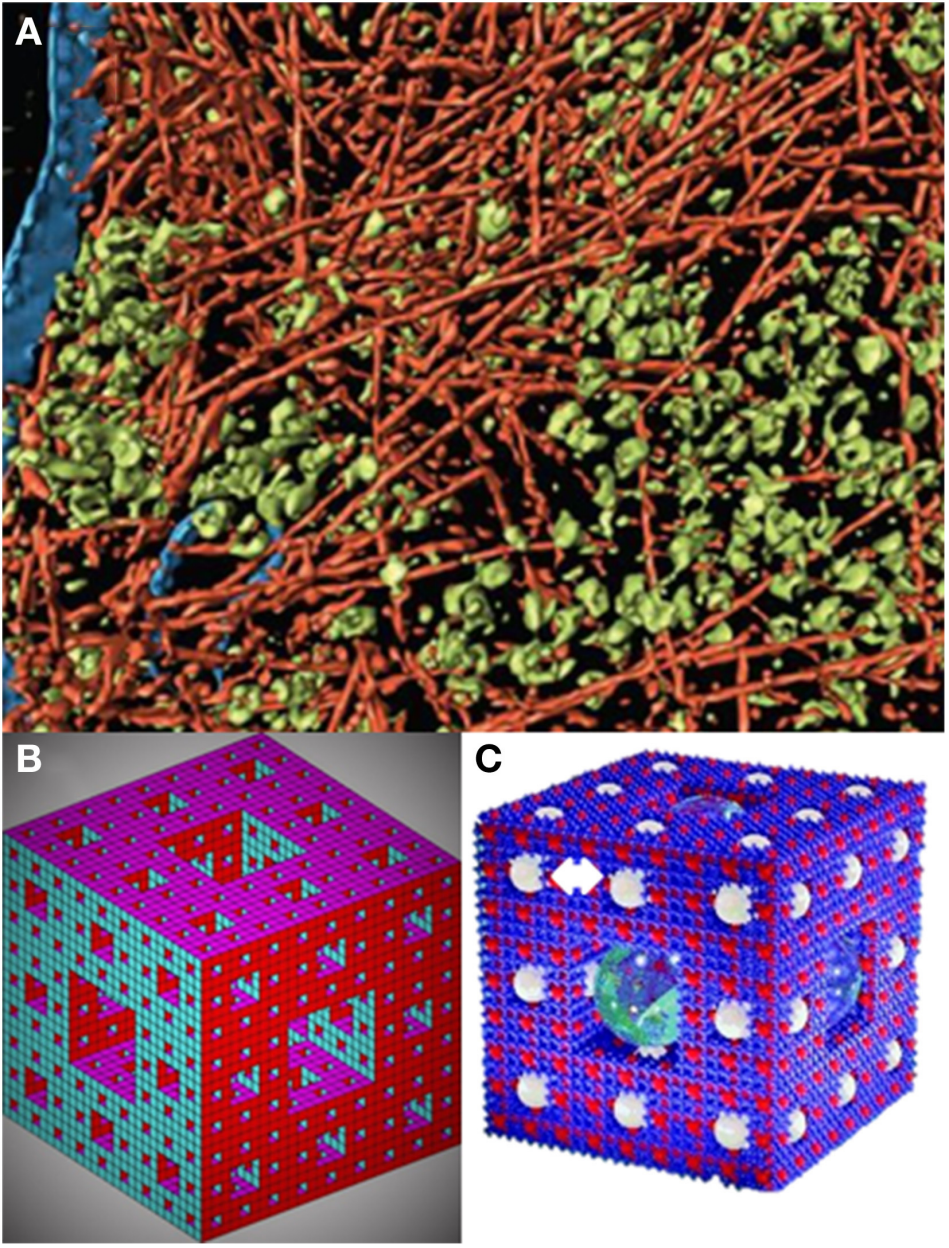
**A fractal sponge-like model of cytoplasmic organization. (A)** Visualization of actin network, membranes, and cytoplasmic macromolecular complexes from electron cryotomography. The pseudo color representation corresponds to actin filaments (red); other macromolecular complexes, mostly ribosomes (green), and membranes (blue) (modified from Medalia et al., 2002). **(B)** The *Sierpinsky carpet* (or gasket) arises from the recursive invariant procedure consisting in repeatedly removing an inverted equilateral triangle from the middle of an initial equilateral triangle (Mandelbrot, 1982). The *Sierpinski–Menger–Mandelbrot sponge* is an extension of the *Sierpinski's carpet* to the three-dimensional Euclidian space (Mandelbrot, 1982) (see also Raicu and Popescu, 2008). This fractal starts from a single cube with an iterative invariant pattern consisting in the removal of a middle cube. The fractal dimension of this structure is 2.727 and if the magnification and removal of the middle cubes continues for *n* → ∞, it is found that the structure becomes a surface packed into a three dimensional Euclidian space (Raicu and Popescu, 2008), or as graphically put by Welch and Clegg (2010): “a fractal geometric form whose progressive ‘fractalization’ results in the surface area increasing to (the theoretical limit of) infinity as the volume shrinks to zero” (Image created by Moses Boone; see http://www.mathworks.com/matlabcentral/fileexchange/3524-sierpinski-sponge). This is what we call the *Sierpinsky–Menger–Mandelbrot sponge*, or **(C**) the version including idealized spheres designated to account for localized metabolic microenvironments (indicated by a double white arrow on the top left), as first proposed by Welch and Clegg (2010) based on a copyrighted image created by Roman Maeder (see http://www.mathconsult.ch/showroom/pubs/MathProg/htmls/p2-16.htm) (modified from Welch and Clegg, 2010).

A model compatible with the 3D visualization of the cellular cytoplasmic organization as a percolation cluster (Aon and Cortassa, 1994; Forgacs, 1995) as suggested by its crowded-organized nature (Medalia et al., 2002) (Figure 2A) is the *Sierpinsky–Menger–Mandelbrot sponge* (Mandelbrot, 1982; Raicu and Popescu, 2008) (Figure 2B) or a modified version introduced by (Welch and Clegg, 2010) to account also for functional aspects given by “confined regions, ranging from organelles to protein complexes…, responsible for the execution of localized metabolic processes…” (Figure 2C).

The modified *Sierpinsky–Menger–Mandelbrot sponge* captures several structural–functional features discussed in this review: (1) the cytoplasmic structure represents a surface with the appearance of a 3D object, because the surface area increases as the volume shrinks. It is straightforward to imagine that the surface area will be modulated by the degree of polymerization of the cytoskeleton in turn influenced by (macro)molecular crowding. (2) It embodies the orderly cytoskeletal organization and the intrinsic randomness of (macro)molecular crowding. These two conditions can create zones of heterogeneity via exclusion (occupied) volume based on attracting (e.g., electrostatic, hydrophobic) or repulsing (e.g., steric) forces. Locally, these regions possess levels of free energy that modulate molecular association, compaction and folding/unfolding of proteins (Zhou et al., 2008; Wirth and Gruebele, 2013). (3) Is a good model for the percolation of fluids through the cytoplasm, including “confined regions” or locally separate clusters of biochemical activity that may extend to other cytoplasmic regions depending on the concentration and status of e.g., enzymatic or organelles' physiology. These localized clusters determine a certain distance with respect to the percolation threshold where local activities become global thus providing a principle of coordinated functional organization (Aon et al., 2004a; Nivala et al., 2012). (4) Enzymes or enzymatic complexes through their binding to the cytoskeleton, and substrates/effectors/messengers percolating through the “sponge” would determine reaction rates (Figure 1) defining local clusters of activity that result in different product concentrations and gradients. (5) It accommodates the existence of “quinary” structures (McConkey, 1982) that drive transiently and loosely grouped ensemble of proteins working together in a dynamic and spatially organized way, e.g., a metabolon or supramolecularly organized enzymatic complex (Srere, 1987), thus explaining compartmentalization in cytoplasmic regions that are not bounded by membranes (Wirth and Gruebele, 2013) (Figure 2C). (6) The sponge-like model of the cytoplasm is sound from the status of intracellular water (Clegg, 1984a,b) and rheological standpoints. The ground plan of living cells has been pictured as a reversible, non-covalent gel network (Luby-Phelps et al., 1986; Rabouille et al., 1990) that can be subjected to sol–gel transitions. A colloidal sol state has liquid properties with well-defined viscosity whereas in a gel viscosity becomes practically infinite with the percolation threshold, given by the concentration of polymers in solution, as the critical parameter at which the sol–gel transitions happen (Forgacs and Newman, 1994).

With a model at hand, we can now put it to test and ask questions and/or verify certain predictions. For example, can confined cytoplasmic clusters of biochemical activity be experimentally demonstrated? Could the same enzyme(s), enzymatic complex or organelle behave differently according to local cytoplasmic conditions, e.g., higher presence of F-actin (G-actin) with respect to microtubules (tubulin)? Can clusters of biochemical activity based on quinary structures be demonstrated, and are these clusters sensitive to the dynamic intracellular environment? Which intracellular conditions define percolation thresholds, and can we show local activity become globally spread? Do these conditions change with cellular stages of growth, division (e.g., G1, S phases) or differentiation? Is the status of cellular cytoplasmic organization different in differentiated with respect to pluripotent stem cells? How, and to what extent, does the intracellular dynamic field of interrelating polymeric forces play out for the dynamics of metabolic and signaling pathways interacting or being influenced by those polymers, or forming supramolecular complexes themselves? All of these are fascinating and relevant questions that now can be addressed experimentally with emerging new methodologies and a unified structural–functional theoretical framework for cytoplasmic organization.

### Conflict of interest statement

The authors declare that the research was conducted in the absence of any commercial or financial relationships that could be construed as a potential conflict of interest.
